# The influence of iris -ciliary angle (ICA) on the vault after implantation of V4c implantable collamer lens: a chain mediation model of ICL haptic related factors

**DOI:** 10.1186/s12886-023-03122-w

**Published:** 2023-10-06

**Authors:** Weina Tan, Zheng Wang, Qingyan Zeng, Xiaohua Lei, Chao Pan, Bao Shu, Lina Jin, Qian Chen

**Affiliations:** 1 Aier Eye Hospital of Wuhan University (Wuhan Aier Eye Hospital), Wuhan, Hubei Province People’s Republic of China; 2 Hankou Aier Eye Hospital, Wuhan, Hubei Province People’s Republic of China; 3 Aier Institute of Refractive Surgery, Guangzhou, Guangdong Province People’s Republic of China; 4 Hongshan Aier Eye Hospital, Wuhan, Hubei Province People’s Republic of China

**Keywords:** Iris -ciliary angle, Implantable collamer lens, Haptic, Ultrasound biomicroscopy, Vault

## Abstract

**Background:**

This study aims to identify the relationship between iris -ciliary angle (ICA) and the vault. Additionally, we also seek to investigate the chain mediating effects of the ICL haptic related factors on this relationship.

**Methods:**

The participants were categorized into three groups according to the ICA value as follows: low ICA group (< 35°); moderate ICA group (35°-70°); high ICA group (> 70°). We compared the preoperative ocular characteristics and postoperative examinations among the three groups. Multiple variable stepwise regression was performed to establish the vault prediction formula. The Process V4.0 in SPSS and Hayes’s PROCESS model 6 was conducted to further elucidate the mediating effects of the final tip point of ICL haptic and the ICL arc-lens arc on the relationship between the ICA and vault.

**Results:**

There was a significant difference in the positions of the ICL haptic among three ICA groups. The regression vault equation was Vault = 679.42–7.26*TCA + 192.30*ACD-196.37*CLR + 73.21* STS(horizontal).A significant negative correlation was found between the ICA and vault (*P* < 0.01).The chain mediation model revealed that the final tip point of ICL haptic and the ICL arc-Lens arc were sequential mediators between ICA and vault (effect = -1.63, 95% CI = -2.72–-0.73).

**Conclusion:**

The ICA was associated with vault via the mediation effect of the final tip point of the ICL haptic and the ICL arc -lens arc. Assessment of ICL haptic related parameters adds significant information to interpret the vault after surgery.

## Background

The EVO Visian Implantable Collamer Lens (ICL), a posterior chamber phakic intraocular lens with a central hole design, has been proven to be a safe and effective vision-correction procedure for people with various degrees of myopia or myopia astigmatism  [1]. Compared to corneal laser surgery, the ICL has multiple advantages such as less dry eye, fewer corneal complications, better visual quality, and a more stable refractive state [2, 3]. Currently, it is considered a viable alternative to corneal laser surgery and is rapidly gaining popularity in patients with low or moderate refractive error [4].

An appropriate vault after ICL implantation is vital to achieve successful long-term outcomes. Generally, an insufficient vault increases the occurrence of anterior subcapsular cataract [5, 6], whereas an excessive vault increases the risk of pigment dispersion and angle closure [7]. Previous studies have recognized that the vault could be affected by a series of anatomical factors including white to white (WTW), anterior chamber depth (ACD), sulcus to sulcus (STS), and crystalline lens rise (CLR) [8–10]. In our recent work, the morphology of the ciliary body was shown to play a crucial role in the vault after ICL implantation. Eyes with the ciliary body in an anterior position were associated with the postoperative high vault [11], while eyes with a posteriorly positioned ciliary body were related to postoperative low vault [12]. However, the mechanism of the influence of the ciliary body on the ICL vault has not been clearly elucidated. An interesting phenomenon was observed in our clinical practice in which the footplates of the ICL were often inserted behind the ciliary body in eyes with a posteriorly positioned ciliary body which resulted in persistent low vault even after exchange to a larger ICL due to low vault during the first postoperative period. Thus, we hypothesized that the position of the ICL haptic may be a key mediator in the association between the morphology of the ciliary body and the vault.

The application of ultrasound biomicroscopy (UBM) in ICL implantation has made it possible to observe the position of ICL haptics in living eyes [13]. ICL implantation has been shown to be diverse in the real world, although the haptics of the ICL are designed to be placed in the ciliary sulcus. Earlier studies have explored the effect of the position of ICL haptics on the vault after ICL implantation [14, 15] and reported that although the haptics in most patients were not placed in the ciliary sulcus, the vault was almost always with in the ideal range. However, the intrinsic correlation between the position of the ICL haptics and the vault remains unknown. Many studies on the subject have only analyzed the position of ICL haptics using subjective and qualitative methods.

The purpose of the current study was to reveal the relationship between the morphology of the ciliary body and the vault after ICL implantation. We also objectively and quantitatively described the characteristics of the ICL haptics using detailed UBM imaging and identified its associated influential factors. Furthermore, we aimed to construct a chain mediation model to demonstrate the chain mediating effects of the ICL haptics positions and ICL haptic related factors on the relationship between the morphology of the ciliary body and the vault.

## Methods

### Patients

This prospective case-series analysis was performed between October 2019 and April 2021 at the Department of Ophthalmology, Hankou Aier Eye Hospital. A total of 181 eyes in 181 patients who had undergone ICL-V4c with at least 3 months of follow-up were enrolled in the study. Only the right eye was selected. The inclusion criteria included age between 18 ~ 45 years, refractive error in the corrected range ( manifest myopia from -0.50 to -18.00 D, astigmatism from 0 to -6.00 D), refractive state stable for at least 1 year, ACD ≥ 2.8 mm, corneal endothelium cell density ≥ 2000 cells/mm^2^, and availability of a high-quality UBM measurements preoperatively and in 3 month postoperatively. Patients with a history of systemic disease, any ophthalmic inflammation, trauma, or previous ocular surgery were excluded. Approval was obtained from the institutional review board. The study was conducted in accordance with the principles of the Declaration of Helsinki and all included patients provided written informed consent.

### Examinations

All participants underwent a thorough anterior and posterior segmental evaluation before surgery, which mainly involved uncorrected and corrected distance visual acuity (UDVA, CDVA), intraocular pressure measurement (IOP), manifest and cycloplegic refractions, slit lamp and dilated fundus examinations, endothelial cell density measurement and A-scan ultrasonography. The axial length (AL) and lens thickness (LT) were obtained from the A-scan report. Indication of horizontal WTW was based on electronic digital caliper. The ACD, anterior chamber angle(ACA), anterior chamber volime(ACV), pupil diameter (PD) was measured using a rotational Scheimpflug system (Pentacam HR Oculus, Wetzlar, Germany).The UBM (Model SW-3200L; Tianjin Suowei Electronic Technology Co, Ltd, Tianjin, China) was performed to obtain the preoperative parameters. Follow-up visits were conducted at 1 day, 1 week, 1 month, 3 months, 6 months and 1 year after surgery, including UDVA, IOP and slit lamp examination for assessment of subjective vault and any adverse effects. Pentacam HR, anterior segment optical coherence tomography (Triton; Topcon Corporation, Tokyo, Japan) and UBM measurement were performed to detect the vault and the position of ICL haptics at 3 months postoperatively.

### Surgery

The implanted ICL sizes were individually determined based on the surgeon’s clinical experience. A total of 131(72.3%) eyes were implanted with the ICL size according to the manufacturer’s recommendations. Twenty-eight (15.5%) eyes were adjusted to a larger size than the WTW-based sizing recommended by the manufacturer, 22 (12.2%) eyes were converted to a smaller ICL size. The ICL V4C implantation procedures were performed by the same experienced surgeon. Cycloplegic and topical anesthetic eye drops were administered 30 min and 5 min before surgery, respectively. After a 3-mm temporal corneal incision was made, the ICL V4C was inserted into the anterior chamber through the incision by an injector cartridge, and the viscoelastic material was injected into the anterior chamber. Next, the four footplates of the ICL V4c were correctly placed in the posterior chamber. The footplates was placed at 2,4, 8 and 10 o’ clock, and the rotation of the ICL or TICL during the surgery was less than 15°.At the end of surgery, the viscoelastic material was completely removed by a balanced salt solution. An antibiotic and steroidal eye drops were administered topically four times daily for 1 week after surgery.

### Definition of UBM variables

UBM was performed under standard illumination (normal room light approximately 150–200 lx) and accommodation conditions (a target was placed on the ceiling for fixation with the other eye). All patients were scanned in a supine position. After topical anesthesia was administered, an appropriately sized plastic eye cup was gently inserted between the eyelids, and the cup was filled with an artificial tear formulation as a coupling medium. Axial and radial scans were taken. The radial scans in the 4 quadrants (superotemporal, inferotemporal, superonasal and inferonasal) displayed the imaging of the iris, ciliary body and anterior surface of the lens preoperatively and the four ICL haptics in the ciliary sulcus postoperatively. To acquire a better depth of field and clearer visualization of the details of the ICL in the axial scans, the probe was held perpendicular to the ocular surface, placed toward the cornea, and moved slightly in the longitudinal position (2–8 o^’^clock or 4–10 o^’^clock) to trace the general condition of the ciliary body and posterior chamber preoperatively or the final tip of the ICL haptics postoperatively. The examiner patiently and meticulously repeated the measurements until the entire continuous posterior chamber or ICL images were obtained with a clear reflectivity of the optical zone, the two final tips and the bend part of the ICL haptics, the scleral spur, the ciliary body, and the anterior surface of the lens.

**Fig. 1:**
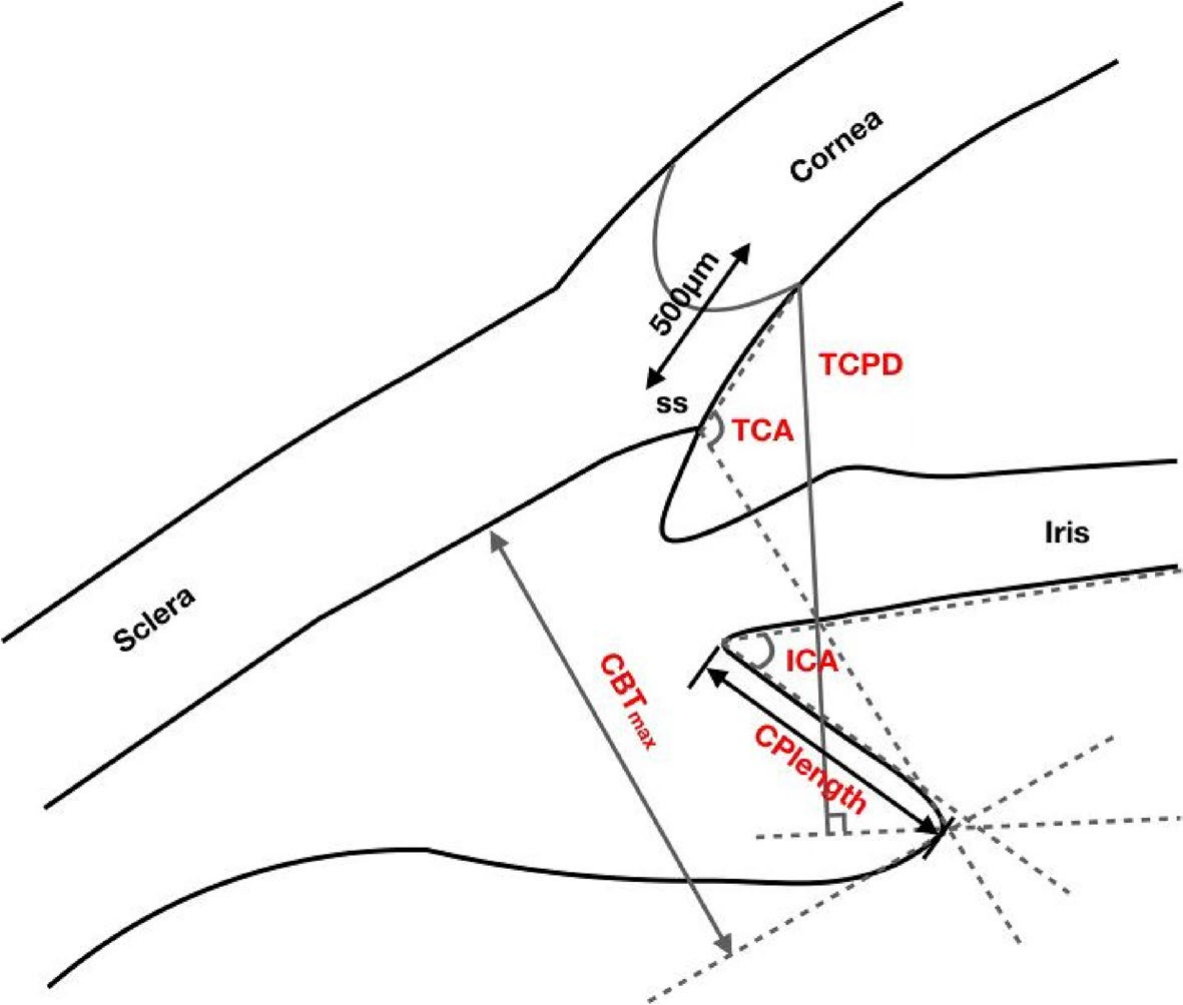
Diagrammatic representation of the UBM measurements of ciliary body parameters. Iris-ciliary angle (ICA) is the angle between the posterior iris surface and the anterior surface of ciliary body; trabecular-ciliary angle (TCA) is measured with the scleral spur as the apex and the corneal endothelium and the anterior surface of ciliary body as the arms of the angle; trabecular ciliary process distance (TCPD) is defined as the length of the line extending from the corneal endothelium 500 μm from the scleral spur perpendicularly to the line which passing through the most inner point of ciliary body and parallel to the iris; maximum ciliary body thickness (CBTmax) is the distance from the most inner point of the ciliary body to the inner wall of scleral or its extended line; ciliary process length (CPlength) was measured as the distance from the inner point of ciliary body to the intersection point of the iris and ciliary body

**Fig. 2:**
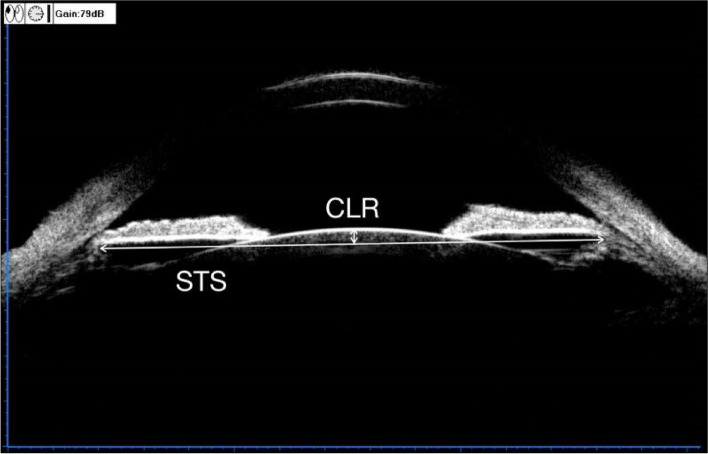
UBM image showing the measurement of the sulcus to sulcus (STS) and the crystalline lens rise (CLR). The CLR was defined as the distance from anterior pole of the lens to STS plane

**Fig. 3:**
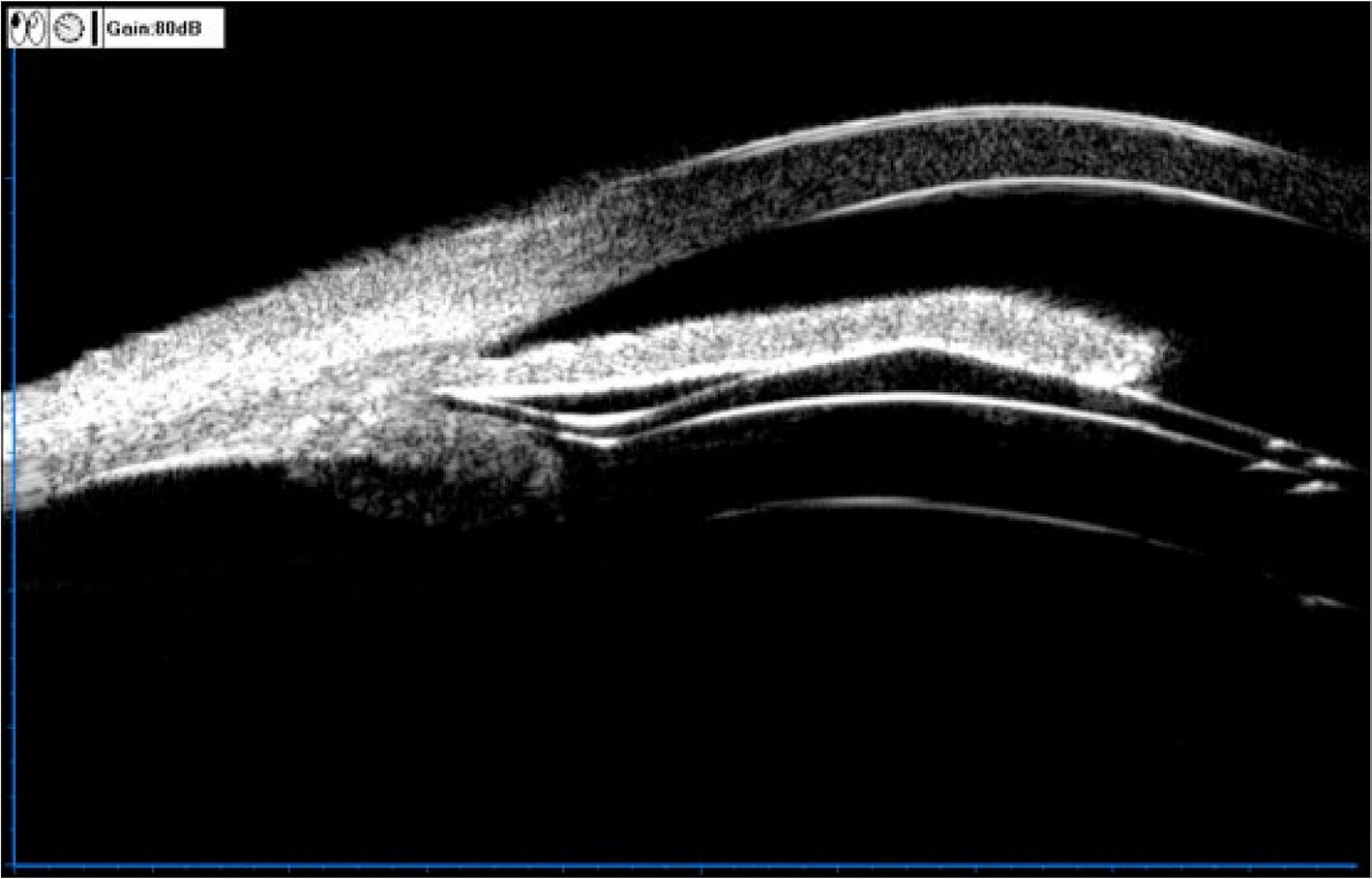
UBM image of the ICL haptic in the ciliary sulcus

**Fig. 4:**
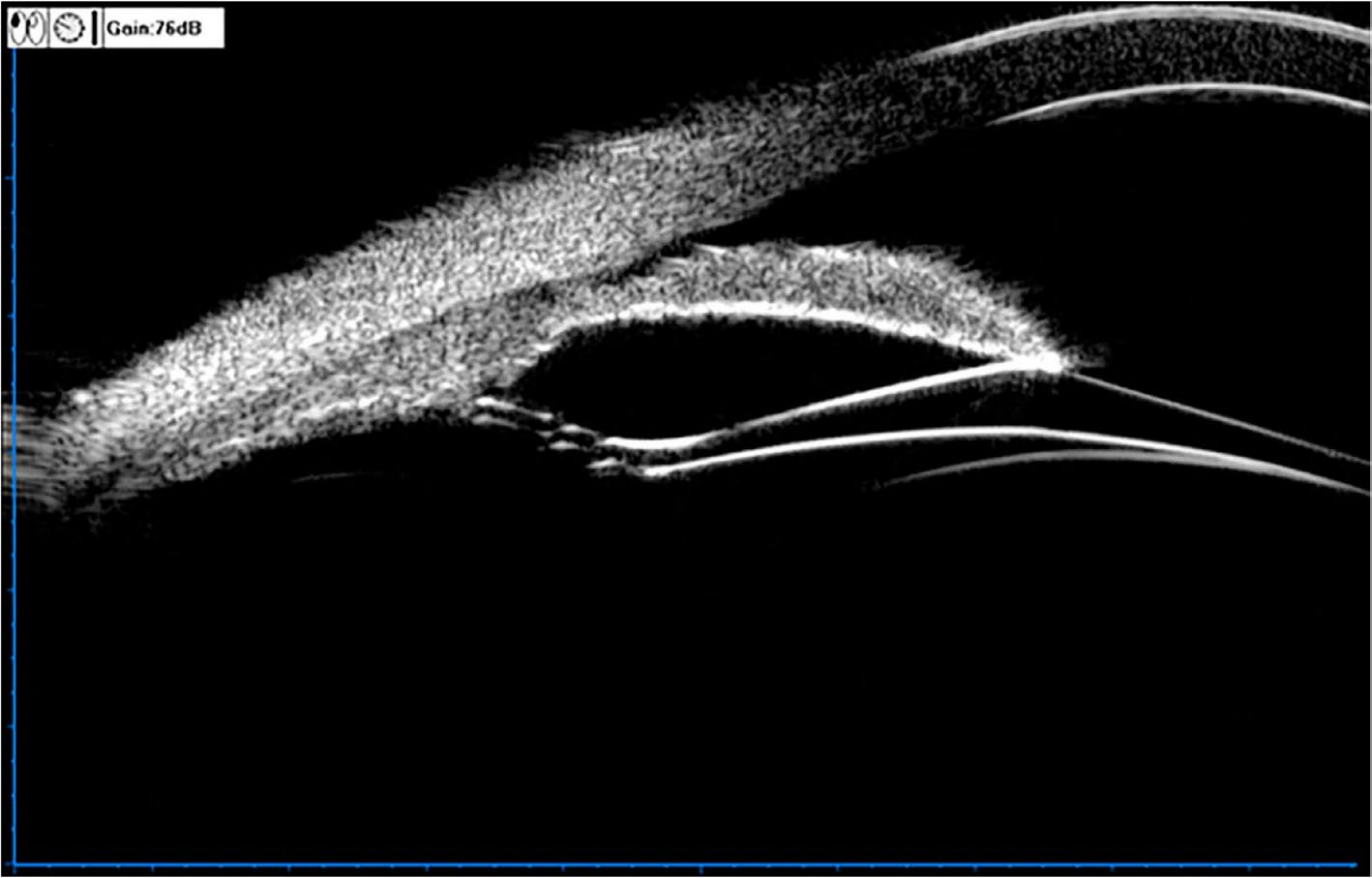
UBM image of the ICL haptic in the ciliary body

**Fig. 5:**
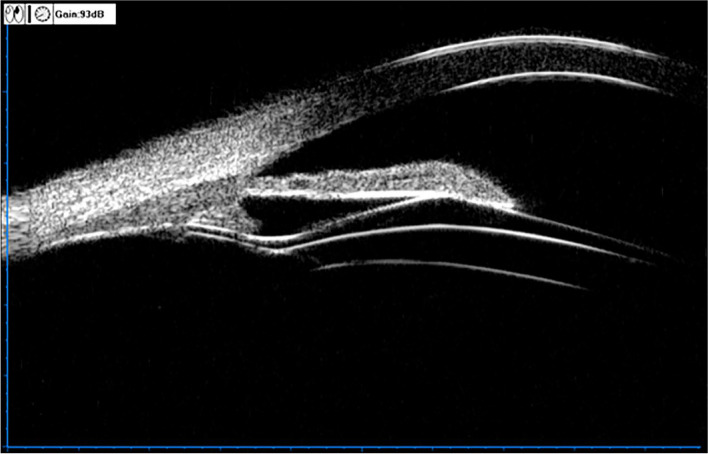
UBM image of the ICL haptic under the ciliary body

**Fig. 6:**
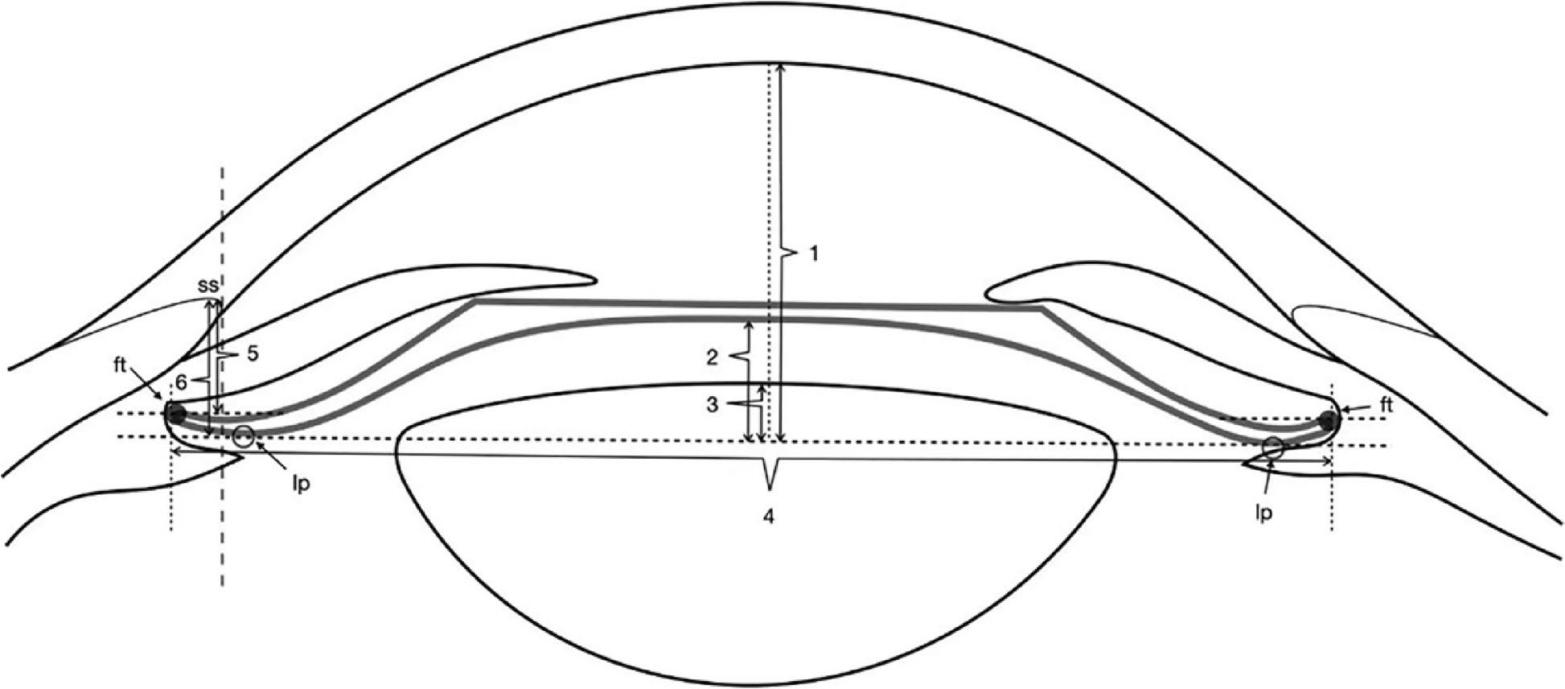
The determination of ICL haptic related parameters on the ultrasound biomicroscopy.1 = Corneal endothelium to ICL haptic (enCornea-ICL haptic) is defined as the length of line extending from the central point of corneal endothelium perpendicularly through ICL haptic plane;2 = The posterior of the ICL to ICL haptic (ICL arc) is the vertical distance from the posterior surface of ICL to the ICL haptic plane; 3 = The height of the crystalline lens from the ICL haptic(Lens arc) is measured from the anterior pole of the lens perpendicularly to the ICL haptic plane; 4 = The ICL haptic diameter (HTH) is the horizontal distance of the two final tip of the ICL haptics; 5 = The final tip of ICL haptic (ftICL haptic) is defined as the perpendicular distance from scleral spur to the parallel line which passing through the final tip of the ICL haptic; 6 = The lowest point of ICL haptic(lpICL haptic) is defined as the vertical distance from scleral spur to the ICL haptic plane; ICL haptic plane is the line which passing through the lowest reflection of two ICL haptics; SS = scleral spur; ft = the final tip of ICL; lp = the lowest point of ICL

The UBM method and related parameter measurements before ICL implantation have been described in detail in previous report [11] (Fig. 1). These measurements include the following: (1) iris-ciliary angle (ICA): the angle between the posterior iris surface and the anterior surface of the ciliary body; (2) trabecular-ciliary angle (TCA): the angle measured with the scleral spur as the apex and the corneal endothelium and the anterior surface of the ciliary body as the arms; (3) trabecular ciliary process distance (TCPD): the length of the line extending from the corneal endothelium at 500um from the scleral spur perpendicularly to the line passing through the innermost point of the ciliary body and parallel to the iris; (4) maximum ciliary body thickness (CBTmax): the distance from the innermost point of the ciliary body to the inner wall of the sclera or its extended line and (5) ciliary process length (CPlength): the distance from the innermost point of the ciliary body to the intersection point of the iris and ciliary body. (6) The CLR, which was defined as the perpendicular distance from the anterior pole of the lens to the horizontal line between the STS distance, was measured on a full view scan from the 3–9 o’clock positions (Fig. 2). According to the average of 3 sequential measurements of preoperative ICA in four quadrants [16], the patients were classified into three groups: low ICA group (< 35°),moderate ICA group (35°-70°) and high ICA group (> 70°). The position of the haptics in relation to the adjacent tissue was assessed by using the radical scanning image. The haptics position was classified as follows:(7) in the ciliary sulcus (Fig. 3); (8) on the ciliary body (Fig. 4); (9) under the ciliary body (Fig. 5). The axial scanning image was measured manually using Image J software. The measured parameters are as follows (Fig. 6):

- (10) Corneal endothelium to ICL haptic (enCornea-ICL haptic): the perpendicular distance from the vertical center of the cornea endothelium to the line that passes through the lowest reflection of two ICL haptics;
- (11) The Posterior ICL to ICL haptic (ICL arc): the perpendicular distance from the posterior reflection of the vertical center of the ICL to the line that passes through the lowest reflection of two ICL haptics;
- (12) The height of the crystalline lens from the ICL haptic (Lens arc):the perpendicular distance from the anterior pole of the lens to the line that passes through the lowest reflection of two ICL haptics;
- (13) The ICL haptic diameter (HTH): the horizontal distance of the two final tips of the ICL haptics;
- (14) The final tip point of ICL haptic (ftICL haptic): the perpendicular distance from the scleral spur to the line that passes through the final tip of the ICL haptic;
- (15) The lowest point of ICL haptic (lpICL haptic): the perpendicular distance from the scleral spur to the line that passes through the lowest point of the ICL haptic.

In all patients, these measurements were performed by one independent well-trained grader in a masked fashion and measured three times. The final tip and lowest point of the ICL haptic were obtained in the four quadrants while other parameters were measured at the two meridians (2–8 o^’^clock and 4–10 o^’^clock) of the central section. The mean value of all measurements in the four quadrants or two meridians was used for analysis.

### Statistical analysis

The data were statistically analyzed using SPSS Statistics (version 25.0, SPSS INC., IBM, USA). The Kolmogorov–Smirnov test was used to assess normal distribution. For normally distributed continuous variables, mean and standard deviation are used to express the data, otherwise, the data are expressed as median (25^th^ and 75^th^ percentile). One-way ANOVA with Bonferroni post-hoc test was used to compare the groups. The Kruskal -Wallis test followed by Bonferroni post hoc test were used to analyze the variables that were not normally distributed. The chi-square test was used to compare the proportion of sex, the ratio of toric-ICL to ICL implanted eyes and ICL haptic status among the three groups. The linear relationship was used to evaluate the correlation between vault and preoperative ocular parameters, Then, multiple variable stepwise regression was performed to assess the association between the vault and preoperative ocular parameters and construct the equation to predict the vault.

Correlations among ICA, the ICL haptic- related parameters and vaults were evaluated using Pearson correlation analysis. In addition to the Pearson analysis, Process V4.0 in SPSS and Hayes’s PROCESS model 6, which allows up to four mediators to be chained in sequence, were used to analyze the mediating effect of the ICA and vault. We conducted a bootstrap method with 1000 repeated samplings to calculate the 95% confidence interval. If the 95% confidence interval contained zero, the indirect effect was not significant. A *P* value below 0.05 was considered statistically significant.

## Results

A total of 181 eyes in 181 patients with ICL V4c implantation were finally enrolled in the study. According to the mean ICA value, the subjects were divided into three groups:27 eyes in the low ICA group, 129 eyes in the moderate ICA group and 25 eyes in the high ICA group. Table 1 provides the demographic and clinical characteristics of the study population. There was no statistical difference among three groups except for ICA, trabecular-ciliary angle (TCA), maximum ciliary body thickness (CBTmax), ciliary process length (CPlength) and trabecular ciliary process distance (TCPD). The mean values of ICA and TCA were lowest in the low ICA group, followed by the moderate and then the high ICA groups. The opposite results were observed for mean CBTmax. The moderate and high ICA groups had a lower CPlength and higher TCPD than the low ICA group.

**Table 1:** Comparison of demographic and preoperative ocular characteristics between eyes with ICL Implantation stratified according to the ICA value

Parameters	Low ICA group	Moderate ICA group	High ICA group	*P* Value
Age	24.18 ± 4.28	25.48 ± 4.99	26.92 ± 5.82	0.15
Sex(male,%)	14.81	26.35	32.00	0.33
T/I(%)	25.92	32.55	20.00	0.40
ICL Size(mm)	12.63 ± 0.38	12.67 ± 0.37	12.65 ± 0.44	0.87
Sph(D)	-7.25(-8.37,-6.25)	-7.50(-8.75,-5.75)	-9.0(-9.62,-6.12)	0.06
Cly(D)	-0.75(-1.0,-0.25)	-0.75(-1.25,-0.50)	-1.25(-1.50,-0.75)	0.14
SE(D)	-8.94(-7.50,-6.37)	-8.06(-9.37,-6.25)	-9.50(-10.43,-7.13)	0.05
IOP(mmHg)	14.51 ± 2.77	14.29 ± 2.65	14.30 ± 2.74	0.92
AL(mm)	26.03 ± 1.48	26.02 ± 1.31	25.95 ± 1.25	0.97
WTW(mm)	11.42 ± 0.42	11.39 ± 0.37	11.27 ± 0.32	0.27
ACD(mm)	3.23 ± 0.28	3.21 ± 0.26	3.14 ± 0.26	0.37
ACA(°)	38.58 ± 3.59	38.64 ± 5.12	37.57 ± 5.19	0.61
ACV(mm^3^)	199.48 ± 38.08	200.90 ± 31.35	194.32 ± 35.46	0.66
PD(mm)	3.26(2.95,3.26)	3.15(2.84,3.15)	3.05(2.86,3.05)	0.54
LT(mm)	3.79 ± 0.22	3.81 ± 0.30	3.91 ± 0.26	0.23
STS_horizontal_ (mm)	11.31 ± 0.60	11.40 ± 0.43	11.33 ± 0.47	0.57
STS_vertical_ (mm)	11.78 ± 0.60	11.75 ± 0.48	11.61 ± 0.49	0.42
ICA(°)	24.31 ± 8.58	51.49 ± 9.60	79.77 ± 9.82	< 0.001^abc^
TCA(°)	104.63 ± 9.56	117.17 ± 10.06	131.55 ± 7.76	< 0.001^abc^
CBTmax (mm)	1.28 ± 0.14	1.17 ± 0.13	1.03 ± 0.13	< 0.001^abc^
CPlength (mm)	0.89 ± 0.14	0.77 ± 0.11	0.76 ± 0.14	< 0.001^ac^
TCPD (mm)	0.94 ± 0.13	1.07 ± 0.12	1.13 ± 0.14	< 0.001^ac^
CLR (μm)	0.41 ± 0.16	0.39 ± 0.22	0.46 ± 0.22	0.24

*T/I* the ratio of toric-ICL to ICL implanted eyes, *ICL Size* the implanted ICL size, *Sph* manifest refractive sphere, *Cly* manifest refractive cylinder, *SE* spherical equivalent, *IOP* intraocular pressure, *AL* axial length, *WTW* horizontal white-to-white diameter, *ACD* anterior chamber depth, *ACA* anterior chamber angle, *ACV* anterior chamber volume, *PD* pupil diameter, *LT* lens thickness, *STS* sulcus-to-sulcus diameter, *ICA* iris-ciliary angle, *TCA* trabecular-ciliary angle, *CBTmax* maximum ciliary body thickness, *CPlength* ciliary process length, *TCPD* trabecular ciliary process distance, *CLR* the crystalline lens rise

^a^ *P* < 0.01for the difference between low ICA and moderate ICA group

^b^ *P* < 0.01 for the difference between moderate ICA and high ICA group

^c^ *P* < 0.01 for the difference between low ICA and high ICA group

A comparison of the ICL haptics position and ICL haptic-related parameters of the study patients is shown in Table 2. The haptics located in the ciliary sulcus occurred most frequently in the low ICA group. The high ICA group had the highest rate of haptics located in the ciliary body compared to the other two groups. The low ICA group had a lower rate of haptics located under the ciliary body compared to the moderate and high ICA groups. The mean ftICL haptic and lpICL haptic were lowest in the low ICA group followed by the moderate and high ICA groups. A statistically significant difference was observed in Lens arc, Lens arc-CLR, ICL arc-Lens arc and vault. We demonstrated that the mean lens arc was lower in the low and moderate ICA groups than in the high ICA group, while the high ICA group had a higher lens arc-CLR and lower ICL arc-lens arc and vault than the low ICA group. SE, LT, WTW, ACD, ACA, ICA,TCA, CBTmax, CPlength, PCA and CLR were associated with the vault in the linear regression analysis, as shown in Table 3. The TCA (Standardized coefficients *β* = -0.38*,P* < 0.001),ACD (*β* = 0.22*,P* < 0.05),LT (*β* = -0.26, *P* < 0.001) and STS(horizontal) (*β* = 0.15, *P* < 0.05) were finally enrolled as the explanatory variables to construct the vault prediction formula in the multiple variable stepwise regression analysis. The vault prediction formula was: vault = 679.42–7.26*TCA + 192.30*ACD-196.37*CLR + 73.21* STS(horizontal).The adjusted R^2^ and D-W value of this prediction formula is 0.32 and 1.98 respectively*,P* < 0.001.Correlations among the ICA, postoperative ICL haptic-related factors and vault are shown in Table 4, and all the variables were significantly correlated (*P* < 0.01). The ICA was positively associated with the ftICL haptic and negatively correlated with the difference between the ICL arc and lens arc as well as the vault (*P* < 0.01). The final tip of the ICL haptic was negatively associated with the difference between the ICL arc and lens arc and the vault. A highly positive significant relationship was found between the difference between the ICL arc and Lens arc and the vault (*P* < 0.01).

**Table 2:** Comparison of the ICL haptic related parameters between eyes with different ICA groups

Parameters	Low ICA group	Moderate ICA group	High ICA group	*P* Value
The haptic in ciliary sulcus (%)	71.29(77/108)	46.51(240/516)	24.00(24/100)	< 0.001^abc^
The haptic in the ciliary body (%)	25.93(28/108)	42.83(221/516)	59.00(59/100)	< 0.001^abc^
The haptic under the ciliary body (%)	2.77(3/108)	10.65(55/516)	17.00(17/100))	0.003 ^ac^
ftICL haptic(mm)	0.76 ± 0.19	0.85 ± 0.16	1.04 ± 0.18	< 0.001^abc^
lpICL haptic (mm)	0.84 ± 0.18	0.93 ± 0.16	1.06 ± 0.17	< 0.001^abc^
lpICL – ftICL(mm)	0.06(0.00,0.11)	0.08(0.00,0.14)	0.03(0.00,0.13)	0.13
HTH (mm) HTH- STS_horizontal_(mm)	11.62 ± 0.45 0.06(-0.33,0.56)	11.63 ± 0.47 0.20(-0.13,0.49)	11.63 ± 0.55 0.32(-0.03,0.58)	0.99 0.43
enCornea-ICL haptic(mm)	1.68 ± 0.27	1.65 ± 0.23	1.72 ± 0.26	0.37
ICL arc(mm)	1.49 ± 0.26	1.45 ± 0.22	1.52 ± 0.26	0.35
Lens arc(mm)	0.89 ± 0.19	0.92 ± 0.19	1.11 ± 0.23	< 0.001^bc^
Lens arc-CLR (mm)	0.44 ± 0.21	0.54 ± 0.22	0.64 ± 0.18	0.003^c^
ICL arc-Lens arc(mm)	0.64 ± 0.34	0.53 ± 0.25	0.41 ± 0.35	0.12^c^
vault(mm)	604.70 ± 230.88	526.93 ± 226.83	409.56 ± 294.90	0.02^c^

*ftICL haptic* the final tip point of ICL haptic, *lpICL haptic* the lowest point of ICL haptic, *lpICL – ftICL* the difference between the lpICL and ftICL, *HTH* The ICL haptic diameter, *HTH- STS*_*horizontal*_ the difference between the HTH and STS, *enCornea-ICL haptic* the corneal endothelium to ICL haptic, *ICL arc* the posterior of the ICL to ICL haptic, *Lens arc* the height of the crystalline lens from the ICL haptic, *Lens arc-CLR* the difference between the lens arc and CLR, *ICL arc-Lens arc* the difference between the ICL arc and Lens arc

^a^ *P* < 0.01 for the difference between low ICA and moderate ICA group

^b^ *P* < 0.01for the difference between moderate ICA and high ICA group

^c^ *P* < 0.01 for the difference between low ICA and high ICA group

**Table 3:** Analysis of the linear relationship of factors associated with vault

Parameters	R^2^	*P* value
SE	0.06	0.01*
IOP	0.01	0.49
AL	0.01	0.20
LT	0.08	0.00*
WTW	0.09	0.00*
ACD	0.05	0.01*
ACA	0.05	0.01*
ACV	0.00	0.70
PD	0.00	0.66
STS(horizontal)	0.01	0.23
STS(vertical)	0.01	0.27
ICA	0.16	0.00*
TCA	0.11	0.00*
CBTmax	0.18	0.00*
Cplength	0.07	0.00*
TCPD	0.01	0.39
PCA	0.06	0.01*
CLR	0.14	0.00*

*SE* spherical equivalent, *IOP* intraocular pressure, *AL* axial length, *LT* lens thickness, *WTW* horizontal white-to-white diameter, *ACD* anterior chamber depth, *ACA* anterior chamber angle, *ACV* anterior chamber volume, *PD* Pupil diameter, *STS* sulcus-to-sulcus diameter, *ICA* iris-ciliary angle, *TCA* trabecular chamber angle, *CBTmax* maximum ciliary body thickness, *CPlength* ciliary process length, *TCPD* trabecular ciliary process distance, *PCA* posterior chamber area, *CLR* crystalline lens rise

^*^p < 0.05

**Table 4:** Correlation among the ICA 、 postoperative ICL haptic related factors and vault

	1	2	3	4
1.ICA	NA			
2.ftICL haptic	0.42**	NA		
3.ICL arc-lens arc	-0.28**	-0.39**	NA	
4.vault	-0.32**	-0.37**	0.85**	NA

*ICA* iris-ciliary angle, *ftICL haptic* the final tip point of ICL haptic, *ICL arc* the posterior of the ICL to ICL haptic, *Lens arc* the height of the crystalline lens from the ICL haptic, *ICL arc-Lens arc* the difference between the ICL arc and Lens arc

^**^ P < 0.01

**Fig. 7:**
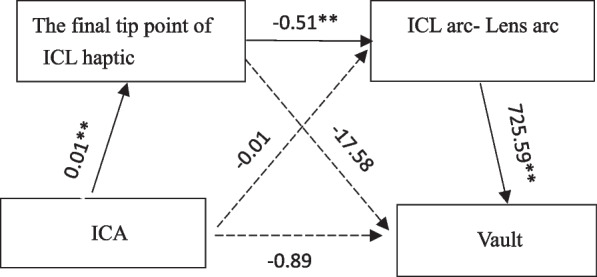
Diagram of the mediator model to explain the association between ICA and clinical vault. Black solid arrow indicates statistically significant direct route, and black dotted arrow represents no statistical significance. * P < 0.05; ** P < 0.01

The mediation effects of the variables on the vault outcomes are displayed in Table 5. There was statistical significance in the total indirect effect, as the 95% CI did not include zero. ICA had no significant indirect effect on the vault via the final tip of ICL haptic or the difference between ICL arc and lens arc alone. ICA had a significant indirect effect on the vault through the chain mediating effect of the final tip of the ICL haptic and the difference between ICL arc and lens arc (effect = -1.63, 95% CI = -2.72–-0.73). Figure 7 shows the results of the mediator models to explain the relationship between the ICA and the vault. Except for the path from the ICA to the vault through the chain of the final tip of ICL haptic and the difference between ICL arc and Lens arc, the other paths in this mediator model were not significant.

**Table 5:** Specific indirect effects of ICA through the final tip point of ICL haptic and ICL arc-lens arcon vault

Effect	Product of coefficients	Bootstrapping 95% BCa confidence interval		
	Point Estimate	SE	Lower	Upper
Total indirect effect	-3.34	1.05	-5.37	-1.19
Path1	-0.08	0.31	-0.77	0.47
Path2	-1.64	0.98	-3.48	0.43
Path3	-1.63	0.52	-2.72	-0.73

Path1: ICA → the final tip point of ICL haptic → vault

Path2: ICA → ICL arc-Lens arc → vault

Path3: ICA → the final tip point of ICL haptic → ICL arc-Lens arc → vault

## Discussion

The current study reported that the ICA was associated with the vault. This finding is consistent with our previous study [11] which revealed that ICA was the major risk factor for excessive vault (> 1000 μm), with every 1-degree reduction in ICA, the risk of excessive vault increased by 4%. Zhu et al. [17] developed a regression equation to predict the vault when testing the accuracy of the formula they found that wide ICA, iris concavity and anteriorly positioned ciliary body may be the reasons for large prediction errors. To our knowledge, the mechanism of the relationship between the ICA and the vault has not been well elucidated, and we hypothesize that ICL haptic-related parameters may play an important role in this relationship. The aim of this study was to describe the characteristics of ICL haptic-related parameters and reveal their effect on the association of the ICA and the vault.

The application of UBM in ICL implantation allows the determination of the exact location of the ICL haptics and its relationship to the surrounding structure. Several studies [13, 15] have reported that the ICL haptics were inserted in different positions and mostly not in the ciliary sulcus as designed. The current study also observed similar results, noting that the ICL haptics placement in the posterior chamber tended to vary. We further quantitatively compared the haptics placement among three different ICA groups. The results showed that the mean ftICL haptic and lpICL haptic were lowest in the low ICA group followed by the moderate and high ICA groups. Considering the flexibility of the ICL material, the ICL haptics may tend to slide down posteriorly in the large angle of the ciliary sulcus with the force of the iris or accommodation. Few studies have examined the association between the position of ICL haptics and the postoperative vault. Shi et al. [18] reported that ICL haptic dislocation is usually related to an inappropriate vault. Zhang et al. [15] observed that the postoperative vault was normal in the majority of 134 eyes despite various positions of ICL haptics. Our study quantitatively analyzed their relationship and found that the ftICL haptic was negatively correlated with the vault.

Other than the positional parameters of the ICL haptics, two more important ICL haptic-related variables (ICL arc and lens arc) were introduced. Theoretically, the ICL arc is composed of the innate vault of the ICL, vertical compression by the iris and the horizontal compression. Prior studies have evaluated the effect of vertical compression on the ICL arc or vault. Batlle et al. [19] used an apparatus to evaluate the effect of horizontal compression on the ICL arc and a found positive association between ICL arc and horizontal compression,with every 1 mm of horizontal compression,the ICL arc increased by a mean of 1100 μm. In daily clinical work and academic research, the disparity between the ICL size and ocular parameters (STS/WTW) is used as an indicator to represent horizontal compression in patients with ICL implantation based on the theory that when the ICL size is larger than the STS, horizontal compression can occur. Kojima et al. [9] developed a vault prediction formula using UBM which indicated that each 0.1 mm of horizontal compression caused a 48μm vault change. Using OCT, Trancon et al. [20] revealed that a 13.7 mm ICL had a larger vault variation for each 0.1 mm of horizontal compression compared to the 12.6 mm and 13.2 mm ICL sizes. However, in an earlier report from Lee et al. [19], only 37% of the eyes achieved the predicted vault when using the horizontal compression model proposed by Batlle. Nam et al. [21] reported no significant difference in postoperative vault between eyes with normal horizontal compression (ICL size larger than the STS) and eyes with low horizontal compression (ICL size smaller than the STS) eyes. Manito et al. [22] demonstrated that the horizontal compression greater than 1.05mm can explain only approximately 75% of the patients who presented with high vault,and low horizontal compression cannot be used to explain low vault. Two questions should be noted when explaining the discrepancy between these studies. The first question involves the evaluation of the horizontal compression. Whether based on WTW or STS, horizontal compression was assessed based on a constant plane. It is important to acknowledge that the “actual STS” and horizontal compression can vary with the ICL haptic plane. Recently, Manito et al. [23] attributed the inter eye differences in vault to the resting position of the ICL haptics, which may be as high as 240 μm. In a new sizing model proposed by Reinstein et al. [24], the ciliary body inner diameter has been shown to be more sensitive in the prediction of vault than the STS, as ICL haptics have been found to rest on the ciliary body in 94% of eyes. The second problem is related to distinguishing between the concepts of ICL arcs and vault. The two conceptions were sometimes chaotic in previous studies. The ICL arc is the whole picture of the ICL in the eyes, while the vault is only one part of the ICL arc, which is meaningful for the clinical work. Our study showed that horizontal compression had a higher correlation with the ICL arc( *r* = 0.26 *P* < 0.001) than the vault( *r* = 0.26 *P* < 0.05).Therefore, the vault may not fully reflect the effect of horizontal compression on the ICL.

The lens arc in our study represents the space occupied by the crystalline lens in the formation of the vault, while previous studies used CLR as a representative measurement. Using UBM, Kojima [9] first introduced the concept of CLR and used it as an explanatory variable to develop a vault prediction formula. Based on the OCT method,the NK formula [25] was constructed using CLR as an independent variable. Gonzalez-Lopez et al. [10] compared the vault in different CLR groups and found that eyes with high vault values tended to have lower CLR values than eyes with low vault values. In the study proposed by Manito [22], which analyzed the risk factors associated with vault, a high CLR was the major contributor to a low vault. Although an increased CLR increased the frequency of low vault in their study, there was a wide range of CLR distributions in the low vault group. The results of our study confirmed that the lens arc(*r* = -0.63, *P* < 0.001)had a stronger correlation with the vault than the CLR(*r* = -0.29,* P* < 0.001). Like the assessment of horizontal compression described above, the CLR measured in all previous studies was based on a constant plane. It is reasonable to conclude that the CLR is nearly equal to the lens arc when the ICL haptic plane is in the ciliary sulcus, whereas it is only a fraction of the lens arc when the ICL haptics rests on or under the ciliary body. This may explain the results proposed by Manito that the eyes with a high CLR had greater chance of presenting a low vault, but a low vault was not always accompanied by a high CLR.

The difference between the ICL arc and lens arc was found to be highly associated with the vault. Our findings are consistent with Reinstein’s report [26] which constructed a trigonometric formula that included the “ICL arc (ICL size-STS)”and the height of the crystalline lens to predict the vault. It is necessary to recognize the vault was only “the tip of the iceberg” and ignored the part of the ICL buried in the crystalline lens. The difference between the ICL arc and lens arc can be helpful to understand the situation of the ICL in the eyes and the formation of the vault.,

This study also advances the literature in relation to the chain mediation effect of the ftICL haptic and ICL arc-Lens arc to explore the mechanism underlying the association between the ICA and the vault. A higher ICA value was associated with a deeper ftICL haptic, which resulted in a deeper ICL haptic plane, which in turn lead to a change in the ICL arc, a higher lens arc and a lower ICL arc -Lens arc, which finally influenced the vault. There is currently a lack of prior research studies in this field, so it is impossible to compare our results to the other studies. Additional studies are required to further explore their relationship in the future.

There are several limitations in the current study. First, there was a relatively small sample size in the low and high ICA groups and a lack of a long follow-up period. A large sample multicenter clinical trial is expected to be carried out in further work. Second, the effect of vertical compression by the iris on the ICL arc was not evaluated. Another limitation concerned image acquisition and software used. The UBM we used only provide the 2D cross-section Imaging, a 3D UBM [27] capable of provide more details of the ICL position with a complete the 360° view and more advanced software enabling the automatic calculation of the ocular parameters is expected to use in further work.

## Conclusions

In conclusion, the present study revealed that preoperative ICA most significantly influenced vault in eyes with ICL implantation. We also provide novel information about the chain mediation role played by the final tip point of the ICL haptic and the difference between the ICL arc and the lens arc in the relationship between the ICA and the vault. The assessment of ICL haptics using UBM is beneficial for the interpretation of the vault.

## Data Availability

All data used or analyzed during this study are available from the corresponding author on reasonable request.

## Abbreviations

ICL: Implantable Collamer lens
WTW: White to white
ACD: Anterior chamber depth
STS: Sulcus to sulcus
CLR: Crystalline lens rise
UBM: Ultrasound Biomicroscopy
UDVA: Uncorrected distance visual acuity
CDVA: Corrected distance visual acuity
IOP: Intraocular pressure
ICA: Iris-ciliary angle
EnCornea-ICL haptic: Corneal endothelium to ICL haptic
ICL arc: The posterior of the ICL to ICL haptic
Lens arc: The height of the crystalline lens from the ICL haptic
HTH: The ICL haptic diameter
FtICL haptic: The final tip point of ICL haptic
LpICL haptic: The lowest point of ICL haptic
TCA: Trabecular-ciliary angle
CBTmax: Maximum ciliary body thickness
TCPD: Trabecular ciliary process distance
LT: Lens thickness
